# Pain relief and associated factors: a cross-sectional observational web-based study in a Quebec cohort of persons living with chronic pain

**DOI:** 10.3389/fpain.2024.1306479

**Published:** 2024-03-15

**Authors:** Meriem Zerriouh, Gwenaelle De Clifford-Faugère, Hermine Lore Nguena Nguefack, M. Gabrielle Pagé, Line Guénette, Lucie Blais, Anaïs Lacasse

**Affiliations:** ^1^ Département des Sciences de la Santé, Université du Québec en Abitibi-Témiscamingue (UQAT), Rouyn-Noranda, QC, Canada; ^2^ Centre de Recherche, Centre Hospitalier de l’Université de Montréal (CHUM), Montréal, QC, Canada; ^3^ Département d’Anesthésiologie et de Médecine de la Douleur, Faculté de Médecine, Université de Montréal, Montréal, QC, Canada; ^4^ Faculté de Pharmacie, Université Laval, Quebec City, QC, Canada; ^5^ Centre de Recherche, Centre Hospitalier Universitaire (CHU) de Québec—Université Laval, Axe Santé des Populations et Pratiques Optimales en Santé, Quebec City, QC, Canada; ^6^ Faculté de Pharmacie, Université de Montréal, Montréal, QC, Canada

**Keywords:** chronic pain, pain, management, relief, treatment, clinical relevance, clinically meaningful, associated factors

## Abstract

**Objectives:**

Randomized clinical trials are used to evaluate the efficacy of various pain treatments individually, while a limited number of observational studies have portrayed the overall relief experienced by persons living with chronic pain. This study aimed to describe pain relief in real-world clinical settings and to identify associated factors.

**Methods:**

This exploratory web-based cross-sectional study used data from 1,419 persons recruited in the community. Overall pain relief brought by treatments used by participants was assessed using a 0%–100% scale (10-unit increments).

**Results:**

A total of 18.2% of participants reported minimal pain relief (0%–20%), 60.0% moderate to substantial pain relief (30%–60%), and 21.8% extensive pain relief (70%–100%). Multivariable multinomial regression analysis revealed factors significantly associated with greater pain relief, including reporting a stressful event as circumstances surrounding the onset of pain, living with pain for ≥10 years, milder pain intensity, less catastrophic thinking, use of prescribed pain medications, use of nonpharmacological pain treatments, access to a trusted healthcare professional, higher general health scores, and polypharmacy. Factors associated with lower pain relief included surgery as circumstances surrounding pain onset, use of over-the-counter pain medications, and severe psychological distress.

**Discussion:**

In this community sample of persons living with chronic pain, 8 out of 10 persons reported experiencing at least moderate relief with their treatment. The analysis has enabled us to explore potential modifiable factors as opportunities for improving the well-being of persons living with chronic pain.

## Introduction

Chronic pain (CP), defined as pain persisting or recurring for more than three months (1), is a serious condition which affects 19% of adults in Canada (2, 3) and has been shown to be a major economic burden (4). It is one of the most common reasons of healthcare utilization (5), a major cause of disability (6), and results in high societal costs (7–9). In Canada, the direct healthcare costs and lost productivity costs of CP are estimated at $40.4 billion per year (10). CP is a complex disease resulting from the interaction of multiple biological (e.g., tissue health, physiology and neurochemistry), psychological (e.g., catastrophic thinking, depression, anxiety), and social (e.g., scepticism, socioeconomic status) factors (11). A multimodal approach is thus recommended to better alleviate CP. Such an approach provides a balance of pharmacologic, physical, and psychological treatments, while building on patient self-management skills (12, 13). Pharmacological treatment may include prescribed and over-the-counter medications such as nonsteroidal anti-inflammatory drugs (NSAIDs), opioids, antidepressants, anticonvulsants, and acetaminophen (14, 15). Physical treatments can be based on a set of stretches and light aerobic exercises performed on a daily basis (e.g., physiotherapy, yoga) or massage therapy (16) and psychological treatments may include cognitive–behavioural therapy, acceptance and commitment therapy, relaxation strategies, and hypnosis (16). Herbal medicine is also used by some patients for pain relief (e.g., St John's Wort, ginger, turmeric) (17).

In real-world clinical settings, the implementation of the multimodal approach is fraught with several challenges, such as difficult access to multidisciplinary pain clinics (18), lack of access to support groups, and poor access to certain nonpharmacological pain treatments for those without insurance and with a limited budget (19). Also, for people living with CP, playing an active role in managing one's disease daily and applying different self-management methods prove challenging. Medications are in fact, used by a majority of persons living with CP (62%–84%) (20–22). Unfortunately, their expectations regarding medication efficacy are often unrealistically high (e.g., expecting complete pain relief), and these expectations are seldom discussed with healthcare professionals to jointly establish realistic and achievable therapeutic goals (23). Moreover, a substantial proportion perceive their treatment as inadequate (37%–64%) (24, 25). The heterogeneity of people living with CP who present with different comorbidities and symptoms of pain may complexify the multimodal approaches proposed by healthcare professionals (26).

Understanding the efficacy of the multimodal approach in real-world clinical settings and unveiling modifiable factors associated with greater pain relief could help provide manageable solutions to alleviate CP and to improve its management, especially for persons without access to facilities offering multidisciplinary care. However, the evidence regarding the efficacy of pain treatments comes mainly from the results of experimental or quasi-experimental clinical trials (14, 15, 24, 25, 27). To our knowledge, a few observational studies representing the real-world have examined self-reported pain relief experienced by people living with CP in the community with their diverse array of treatments (24, 25). Those studies measured the relief provided by treatments independently of pain intensity, but have used simple dichotomous measurements—e.g., perceived treatment adequacy (adequate/inadequate) (25) or pain control from medication (adequate/inadequate) (24)—to evaluate pain relief. A more discriminative portrait is thus warranted. In addition, data is scarce about factors associated with pain relief using multivariable analysis in order to account for and understand the simultaneous effects of multiple variables. Furthermore, these studies were conducted in Asian or European countries (24, 25), potentially not reflecting the reality of pain treatment in Canada. The present study thus aimed to provide a detailed depiction of the pain relief experienced by persons living with CP in real-world clinical settings. Sociodemographic and clinical factors associated with moderate to substantial (30%–60%) or extensive pain relief (70%–100%), as opposed to minimal pain relief (0%–20%), were also explored.

## Methods

### Study design and data source

This cross-sectional observational study was conducted using the ChrOnic Pain trEatment (COPE) Cohort (28) data. It consists of a database containing information about 1,935 persons living with CP, collected through a web-based cross-sectional survey conducted between June and October 2019. The COPE Cohort was established to better understand the real-life use of pharmacological and nonpharmacological treatments among people living with CP in the province of Quebec (Canada) (28). Participant selection criteria were as follows: (1) reporting living with persistent or recurrent pain for more than three months [as per ICD-11 definition (1)]; (2) aged 18 years or older; (3) residing in Quebec; and (4) ability to complete an online questionnaire in French. The Cohort methods are described in detail elsewhere (28). The COPE Cohort profile was found to be comparable to random samples of Canadians living with CP in terms of age, employment, education and pain characteristics. However, women are overrepresented [84% vs. 55%–65% in Canadian CP samples (28)], justifying gender-stratified or gender-standardized statistics and multivariable analyses. The study protocol was approved by the Research Ethics Committee of the University of Quebec in Abitibi-Témiscamingue (#2018-05-Lacasse, A.) and all participants gave their informed electronic consent. No monetary or material incentive was given for participation. The present study used self-reported data from participants who answered the questionnaire section about pain relief (*n* = 1,419).

### Questionnaire and variables

The web-based questionnaire consisted of items used in prior studies and validated composite scales (variables and the integral questionnaire are presented in Supplementary Appendix S1). The questionnaire items were derived from recommended outcome domains and core measures: the Initiative on Methods, Measurement, and Pain Assessment in Clinical Trials (IMMPACT) (29, 30), the Canadian minimum dataset for chronic low back pain research (31), as well as measures included in the Quebec Pain Registry (32). In addition to the variables prioritized by the research team (the balance between validity and parsimony was thoroughly assessed), all indicators identified as a minimum dataset by the Canadian Registry Working Group of the Strategy for Patient-Oriented Research Chronic Pain Network (SPOR CPN) (33) were included in the questionnaire: pain location, circumstances surrounding onset, duration, frequency, intensity, neuropathic component, interference, physical function, anxiety and depressive symptoms, age, gender, and occupational status.

#### Pain relief

Participants were invited to rate their pain relief using a 0 to 100 scale, expressed as percentages with 10-unit increments, where 0 represented no relief, and 100 represented complete relief. For this purpose, the following question was asked immediately after thoroughly inquiring about participants' pharmacological and nonpharmacological pain treatments: “Overall, how much relief do you get from the treatments or medications currently used in your treatment?”. The format of the response choices was inspired by the pain relief question from the Brief Pain Inventory (BPI) (34), which typically measures the relief provided by treatments over the last 24 h. We then chose to analyze this measure in the form of clinically meaningful categories rather than on a continuous scale to enhance the interpretability of the results. According to the literature, pain relief can be considered moderate and clinically meaningful when ≥30%, substantial when ≥50% and extensive when ≥70% (35, 36). According to the distribution of our data, three groups were thus created to classify the dependent variable: (1) minimal pain relief (0%–20%), (2) moderate to substantial pain relief (30%–60%) and (3) extensive pain relief (70%–100%).

#### Factors potentially associated with greater pain relief and covariables

The inclusion of a wide range of variables within the COPE Cohort facilitated a comprehensive selection of potential sociodemographic and clinical factors. The identification of relevant variables was guided by two models for reference. The first was the Andersen model (37), which is widely used in healthcare studies (38, 39). It focuses on factors influencing healthcare utilization, such as predisposing factors (e.g., gender, age, education level, lifestyle), facilitating/inhibiting factors (e.g., region of residence, having a family physician), and need factors (e.g., self-reported symptoms, perceived general health). The second was the biopsychosocial model of CP (40) according to which biological, psychological, and social factors interact with the nervous system and impact the onset and experience of pain. Consequently, the present study incorporated a diverse set of sociodemographic, psychosocial, and clinical variables. Sociodemographic characteristics, included age, gender identity, stereotypically feminine and masculine personality traits according to the Bem Sex-Role Inventory (BSRI) (41, 42), professional status, education level, country of birth, living in a remote area, and receiving disability benefits. CP characteristics included circumstances surrounding its onset (semi-closed question e.g., accident, disease, stressful event), multisite pain (≥2 pain sites assessed using a semi-closed question listing 21 bodily locations), pain frequency (continuous/recurrent), pain duration, pain intensity in the past seven days and at its worst (0–10 numerical rating scale), neuropathic pain screening using the DN4 (Douleur Neuropathique en 4 Questions) (43), pain interference according to the Brief Pain Inventory (44), and agreeing with the statement “I feel that my pain is terrible and it's never going to get any better” (this single item from the Pain Catastrophizing Scale (45) is referred to as “catastrophizing” in the National Institute of Health [NIH] minimal dataset for chronic low back pain (46) and in the STarT Back Screening Tool (47)). Pain management assessment included dichotomized measures (yes/no) of the use of prescription or over-the-counter medications, nonpharmacological treatments, and access to a trusted health professional for pain management. Were also measured psychological distress (Patient Health Questionnaire-4 [PHQ-4] (48)), perceived general health (12-Item Short Form Survey version 2 [SF-12v2] subscale (49)), physical functioning [SF-12v2 subscale (49)], the number of medications currently used (all health conditions considered; polypharmacy was defined as the use of ≥5 medications), feeling the need to reduce alcohol or drug consumption, cigarette smoking, and cannabis use for pain management.

### Statistical analysis

The characteristics of participants were depicted using descriptive statistics (numbers and proportions for categorical variables; means, standard deviations, medians and interquartile ranges for continuous variables). To complete the first objective (depiction of the pain relief), the percentage of participants reporting each 10-unit increment on the 0%–100% pain relief scale were reported for the entire sample and according to pain intensity and gender identity as per good practices in terms of sex- and gender-based analysis. Stratification was also achieved according to nonpharmacological treatments used by participants. To achieve the study's second objective (identify sociodemographic and clinical factors associated with greater pain relief), a multivariable multinomial regression model was used (50). In the model, minimal pain relief (0%–20%) was used as the reference group, to which the moderate to substantial pain relief group (30%–60%) and the extensive pain relief group (70%–100%) were compared. All the sociodemographic and clinical factors were included in the model. The *a priori* selection of those variables was based on the latest recommendations (51). Due to our substantial sample size, this method was favoured over criticized selection techniques such as relying on bivariate regression analysis *p*-values (51) or stepwise selection (50). Variance inflation factors (VIFs) below 5 were used to detect potential multicollinearity issues (52). The adjusted odd ratios (aOR) estimating the associations between independent variables and the likelihood of getting moderate-substantial or extensive pain relief (vs. minimal pain relief), along with their 95% confidence intervals (95% CIs) and *p*-values were computed. A sensitivity analysis was carried out to assess the impact of a multiple imputation technique for missing data (50) on our conclusions. Multiple imputation by fully conditional specification (FCS) method was applied with 5 repetitions. The Hosmer-Lemeshow test (53) confirmed goodness of fit of the models (Chi-square:12.2–18.7; *p* = 0.3–0.7). All analyses were achieved using SPSS Statistics for Windows version 27 (IBM Corp., Armonk, NY, USA) and SAS version 9.4 (SAS Institute, Cary, NC, USA).

## Results

### Participant characteristics

Among the 1,935 participants of the COPE Cohort, 1,419 persons (73.3%) answered the question about the percentage of pain relief brought by the treatments or medications currently used. No clinically important differences were observed between those included and excluded (*n* = 516) in terms of the proportion of individuals born in Canada (95.9% vs. 92.9%), having a post-secondary education (79.2% vs. 76.9%), or residing in remote regions (23.9% vs. 18.8%). However, a higher proportion of women (84.0% vs. 63.2%) were included, justifying the use of gender-stratified or gender-standardized statistics and multivariable analyses. The mean age was slightly lower among included participants (50 vs. 57 years old).

Participant characteristics are reported in Table 1. Regarding pain characteristics, average pain intensity in the past 7 days (0–10 numerical rating scale) was 5.4 ± 1.9, and more than half of the participants (51.8%) reported living with pain for ≥10 years. Prescribed pain medications were used by 79.9% of participants, over-the-counter pain medication by 67.0% and nonpharmacological approaches (physical or psychological treatments) by 85.9%. The perceived general health SF-12v2 score in our sample was 36.4 ± 12.7 [scores <47 indicate impaired wellbeing when compared to general population norms (49)].

**Table 1:** Sample characteristics.

Characteristics^a^ (*n* = 1 419)	No. (%) of participants^b^	
Sociodemographic profile		
Age (years)—mean ± SD	49.74	±13.25
Gender identity		
Women	1,173	(83.97)
Men	220	(15.75)
Non-binary	4	(0.28)
Country of birth		
Canada	1,324	(95.94)
Other	56	(4.06)
Indigenous self-identification		
Yes	25	(1.84)
No	1,335	(98.16)
Race self-identification		
White	1,343	(97.95)
Other	28	(2.04)
Employment		
Worker	502	(36.38)
Unemployed^c^	878	(63.62)
Education level		
Post-secondary education	1 090	(79.21)
No post-secondary education	286	(20.78)
Living in remote region^d^		
Yes	330	(23.86)
No	1,053	(76.14)
Pain characteristics		
Multisite pain (≥2 sites)		
Yes	1,264	(89.08)
No	155	(10.92)
Generalized pain		
Yes	506	(35.66)
No	913	(64.34)
Most common pain locations^e^		
Back	885	(62.37)
Neck	638	(44.96)
Shoulders	621	(43.76)
Legs	546	(38.48)
Hips	536	(37.77)
Pain frequency		
Continually	1,231	(87.12)
Occasionally	182	(12.88)
Pain duration (years)		
<10	682	(48.17)
≥10	734	(51.84)
Pain intensity		
On average in the past 7 days (0–10)—mean ± SD	5.43	±1.94
At its worst in the past 7 days (0–10)—mean ± SD	7.28	±1.75
Mild (scores 1–4/10)	440	(31.38)
Moderate (scores 5–7/10)	753	(53.71)
Severe (scores 8–10/10)	209	(14.91)
Neuropathic pain		
Yes	754	(53.25)
No	662	(46.75)
Pain treatment		
Current use of prescribed medications		
Yes	1,131	(79.93)
No	284	(20.07)
Current use of over-the-counter pain medications		
Yes	949	(67.02)
No	467	(32.98)
Current use of nonpharmacological pain treatments		
Yes	1,215	(85.88)
No	200	(14.12
Health profile		
General Health (SF-12 v2 score)—mean ± SD	36.4	±12.7

NRS, numerical rating scale; SD, standard deviation; SF-12, 12-item short form survey version 2.

^a^ Proportion of missing data across presented variable ranges between 0 and 4.46%.

^b^ Unless stated otherwise.

^c^ Including retired, unemployed.

^d^ Remote resource regions as defined by Revenu Quebec (i.e., the provincial revenue agency): Bas-Saint-Laurent, Saguenay–Lac-Saint-Jean, Abitibi- Témiscamingue, Côte-Nord, Nord-du-Québec, Gaspésie–Îles-de-la-Madeleine. Non-remote regions are near a major urban center.

^e^ Categories are not mutually exclusive.

### Percentages of pain relief

**Figure 1:**
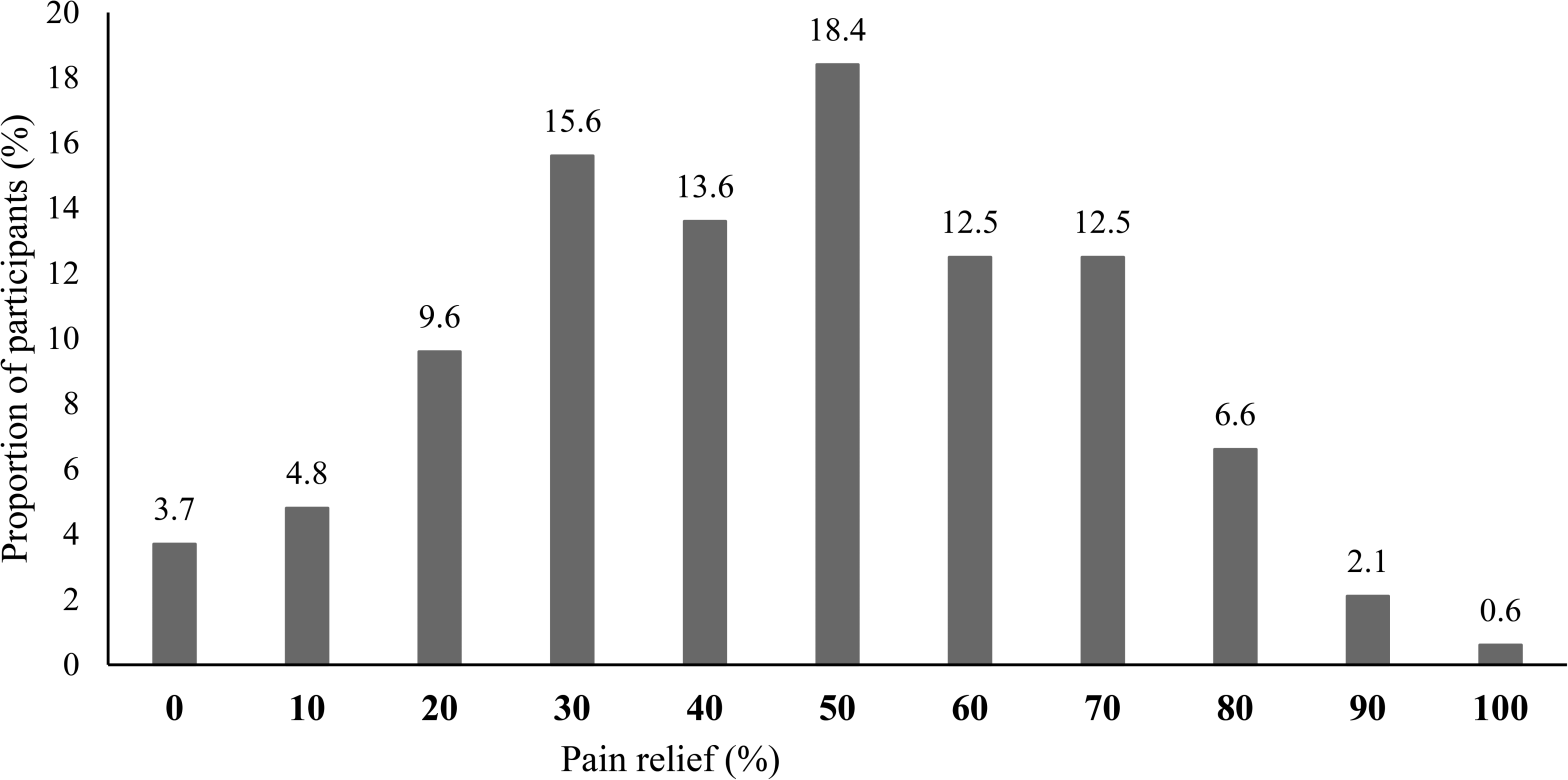
Percentages of participants reaching each 10-unit increment on the 0%–100% pain relief scale (*n* = 1,419). Mutually exclusive categories (total equals 100%).

**Figure 2:**
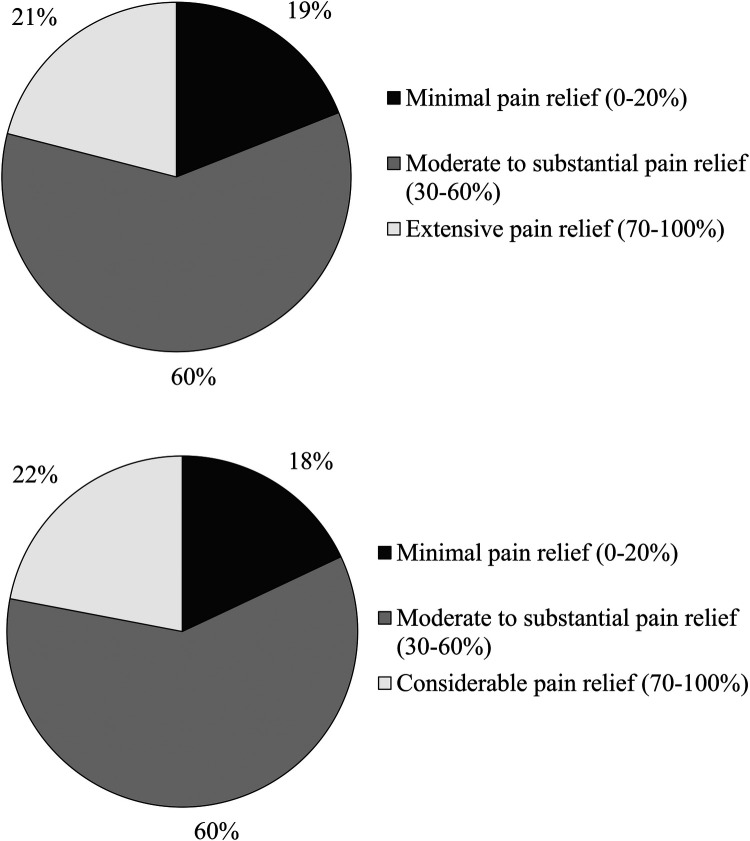
Percentages of participants reaching various cut-offs of pain relief (*n* = 1,419). The upper panel shows results among the woman subsample and lower panel among the men subsample.

**Figure 3:**
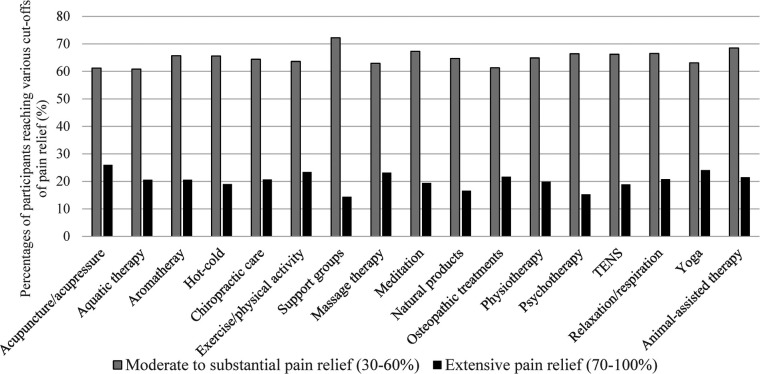
Percentages of participants reaching various cut-offs of pain relief (*n* = 1,419) among users of various physical and psychological treatments. Non-mutually exclusive categories of treatment users. TENS = Transcutaneous electrical nerve stimulation; For statistically sound numbers, achievement of pain relief cut-offs are presented for treatments used by at least 100 participants. For statistically sound numbers, achievement of pain relief cut-offs is presented for treatments used by at least 100 participants. Less than 100 participants used biofeedback, ergotherapy, hypnosis, neurostimulator implantation, group intervention, music therapy, homeopathic product, virtual/augmented reality, reflexology, reiki, tai chi or taping/elastic-bands.

The percentages of participants reaching each 10-unit increment on the 0%–100% pain relief scale are presented in Figure 1. Minimal pain relief (10%–20%) was reported by 18.2% of participants, moderate to substantial pain relief (30%–60%) by 60.0% of participants, and extensive pain relief (70%–100%) by 21.8% of participants. In other words, 81.8% reached the 30% clinically meaningful pain relief cut-off suggested in the literature (35). The proportions of participants reaching such a pain relief cut-off among persons reporting mild, moderate and severe pain intensity were respectively 89.5%, 81.3%, and 68.4%. Figure 2 shows the percentage of participants reaching a particular percentage of pain relief in participants who self-identified as women and men respectively, the portrait being very similar. In the non-binary group (*n* = 4), three persons experienced moderate to substantial (30%–60%) pain relief, and one reported extensive (70%–100%) pain relief. Percentages of participants reaching various cut-offs of pain relief among users of various nonpharmacological treatments (physical and psychological) are presented in Figure 3.

### Factors associated with pain relief

Table 2 shows the estimates of the multivariable multimodal regression model used to investigate the relationship between the different factors and moderate to substantial (30%–60%) or extensive pain relief (70%–100%). Factors associated with an increased likelihood of being in the moderate to substantial pain relief group (30%–60%), as compared to the minimal pain relief group (0%–20%), were: (1) the use of prescribed pain medications (aOR = 2.2, 95% CI: 1.4–3.5), (2) the use of nonpharmacological pain treatments (aOR = 2.2, 95% CI: 1.3–3.5), (3) self-reported access to a trusted healthcare professional for pain management (aOR = 1.5, 95% CI: 1.0–2.2), and (4) use of five or more drugs (polypharmacy) (aOR = 1.8, 95% CI: 1.2–2.8). In contrast, factors associated with a decreased likelihood of reporting moderate to substantial pain relief were: (1) greater pain intensity (moderate vs. mild pain intensity; aOR = 0.3, 95% CI: 0.2–0.6), and (2) feeling that pain is terrible and will never improve (aOR = 0.6, 95% CI: 0.4–0.9).

**Table 2:** Factors associated (*p* < .05) with greater pain relief in the multivariable multinomial regression model.

Variables	Reference: Pain relief 0%–20%	Adjusted odds ratios^a^	95% confidence interval		*p*-value
Chronic pain characteristics					
Circumstances surrounding pain onset reported to be surgery (yes/no)	30%–60%	0.773	0.459	1.300	0.3311
	70%–100%	**0**.**490**	**0**.**245**	**0**.**979**	**0**.**0435**
Circumstances surrounding pain onset reported being related to a stressful event (yes/no)	30%–60%	1.479	0.900	2.431	0.1223
	70%–100%	**2**.**124**	**1**.**161**	**3**.**884**	**0**.**0145**
Duration of pain					
5–9 years (vs. <5 years)	30%–60%	1.505	0.913	2.480	0.1086
	70%–100%	1.480	0.794	2.759	0.2177
≥10 years (vs. <5 years)	30%–60%	1.145	0.746	1.758	0.5353
	70%–100%	**1**.**730**	**1**.**014**	**2**.**950**	**0**.**0442**
Pain intensity					
Severe/8–10 NRS scores (vs. mild/1–4 NRS scores)	30%–60%	0.615	0.377	1.002	0.0512
	70%–100%	**0**.**375**	**0**.**214**	**0**.**659**	**0**.**0006**
Moderate/5–7 NRS scores (vs. mild/1–4 NRS scores)	30%–60%	**0**.**298**	**0**.**154**	**0**.**575**	**0**.**0003**
	70%–100%	**0**.**167**	**0**.**071**	**0**.**392**	**<**.**0001**
Feeling that pain is terrible and it's never going to get any better (yes/no)	30%–60%	**0**.**579**	**0**.**370**	**0**.**905**	**0**.**0166**
	70%–100%	**0**.**387**	**0**.**228**	**0**.**656**	**0**.**0004**
Pain treatment					
Use of prescribed pain medications (yes/no)	30%–60%	**2**.**168**	**1**.**359**	**3**.**460**	**0**.**0012**
	70%–100%	**2**.**275**	**1**.**274**	**4**.**063**	**0**.**0054**
Use of over-the-counter pain medications (yes/no)	30%–60%	0.973	0.669	1.413	0.8842
	70%–100%	**0**.**582**	**0**.**372**	**0**.**912**	**0**.**0182**
Use of nonpharmacological pain treatments (yes/no)	30%–60%	**2**.**172**	**1**.**336**	**3**.**532**	**0**.**0018**
	70%–100%	0.652	0.360	1.181	0.1581
Reporting having access to a trusted healthcare professional for pain management (yes/no)	30%–60%	**1**.**500**	**1**.**006**	**2**.**235**	**0**.**0464**
	70%–100%	**2**.**578**	**1**.**480**	**4**.**488**	**0**.**0008**
Health profile and lifestyle					
Psychological distress according to the PHQ-4					
Mild (vs. none)	30%–60%	1.075	0.636	1.819	0.7867
	70%–100%	0.952	0.527	1.719	0.8705
Moderate (vs. none)	30%–60%	1.085	0.604	1.948	0.7849
	70%–100%	0.695	0.348	1.390	0.3039
Severe (vs. none)	30%–60%	0.574	0.307	1.072	0.0814
	70%–100%	**0**.**320**	**0**.**143**	**0**.**716**	**0**.**0056**
General Health (SF-12 v2 score)	30%–60%	1.010	0.993	1.027	0.2376
	70%–100%	**1**.**033**	**1**.**012**	**1**.**055**	**0**.**0021**
Polypharmacy (≥5 medications; yes/no)	30%–60%	**1**.**826**	**1**.**177**	**2**.**832**	**0**.**0072**

Bold text indicates a statistically significant association (*p* < .05).

NRS: Numerical rating scale; PHQ-4: 4-item Patient Health Questionnaire; SF-12: 12-Item Short Form Survey version 2.

^a^ The table only shows variables with a statistically significant association with the outcome. The multivariable analysis was adjusted for the following covariables: circumstances surrounding pain onset (accident, repetitive movements, cancer, non-cancerous disease, other, no precise circumstances, unknown), multisite pain, pain frequency, neuropathic component, pain interference, age, gender-stereotyped personality traits, country of birth, employment, working full- or education level, living in a remote region, receiving disability benefits, physical functioning, excessive polypharmacy (≥10 medications), feeling the need to reduce alcohol or drug consumption, cigarette smoking, cannabis use for pain management.

Factors associated with an increased likelihood of being in the extensive pain relief group (70%–100%), as compared to the minimal pain relief group (0%–20%), were: (1) reporting a stressful event as circumstances surrounding pain onset (vs. no such circumstances; aOR = 2.1, 95% CI: 1.2–3.9), (2) living with CP for ≥10 years (aOR = 1.7, 95% CI: 1.0–3.0), (3) using prescribed pain medications (aOR = 2.3, 95% CI: 1.3–4.1), (4) reporting having access to a trusted healthcare professional for pain management (aOR = 2.6, 95% CI: 1.5–4.5), and (5) having high general health scores (OR = 1.0, 95% CI: 1.0–1.1, i.e., a clinically meaningful 10-point increase in this continuous variable leads to a 10.3 fold increase in the odds). On the other hand, they had fewer chances of: (1) reporting surgery as circumstances surrounding pain onset (vs. no such circumstances; aOR = 0.5, 95% CI: 0.2–1.0), (2) reporting moderate (OR = 0.2, 95% CI: 0.1–0.4) or severe (aOR = 0.4, 95% CI: 0.2–0.7) pain intensity, (3) feeling that pain is terrible and will never going to get any better (aOR = 0.4, 95% CI: 0.2–0.7), (4) using over-the-counter pain medications (aOR = 0.6, 95% CI: 0.4–0.9), and (5) reporting severe psychological distress (vs. mild psychological distress; aOR = 0.3, 95% CI: 0.1–0.7). Multiple imputations of missing values did not change these conclusions.

## Discussion

This study aimed to describe the pain relief brought by treatments used in real-world clinical settings and, in an exploratory fashion, to identify clinical, psychosocial, and sociodemographic factors associated with greater pain relief. Among our participants, 81.8% reported experiencing at least moderate pain relief. Various factors were found to be significantly associated with greater pain relief, including reporting a stressful event as a circumstance surrounding the onset of pain, living with pain for ≥10 years, milder pain intensity, less catastrophic thinking, use of prescribed pain medications, use of nonpharmacological pain treatments, access to a trusted healthcare professional, higher general health scores and polypharmacy. Factors associated with lower pain relief included surgery as circumstances surrounding pain onset, use of over-the-counter pain medications, and severe psychological distress.

### Percentages of pain relief

Some cross-sectional observational studies have measured pain relief experienced by persons living with CP (24, 25). However, such studies have used poorly discriminating dichotomous measures despite the recommendation by IMMPACT to measure pain relief percentages, independently from pain intensity, along with the improvement of physical and emotional functioning (29, 30, 35). In the present study, only 18.2% of participants reported minimal pain relief (0%–20%), as opposed to two large studies conducted in over 25 countries and regions across Europe and Asia who showed that 37%–64% of the participants reported treatment inadequacy/inadequate pain control from medications (yes/no) (24, 25). A significant proportion of our participants reported at least moderate relief (≥30%), specifically 81.8%. This finding is surprising given the above-mentioned studies and literature highlighting poor physical and psychological health among persons living with CP (24, 25). This could be attributed to our web-based community sample, as opposed to several CP studies conducted within pain clinic settings with more severe populations. Indeed, in comparison to a population of individuals living with CP followed in tertiary care in the province of Quebec (32), our study population had a lower average pain intensity in the past seven days (5.4 ± 2 vs. 6.7 ± 2), a lower proportion of individuals with evidence of neuropathic pain (53% vs. 76%), and a lower proportion of individuals reporting pain interference with general activity (BPI item ≥7/10: 51.4% vs. 59.17%). Our sample also had a higher proportion of individuals living with pain for ≥10 years (51.8% vs. 26.2%), and possibly more time to find better ways to manage their pain. It is a positive observation to have as many participants reporting at least moderate relief. Such a result suggests the importance of future research targeting samples that are more representative of the general population of persons living with CP, including individuals experiencing better pain relief. Those persons can certainly provide valuable insights into their pain management strategies.

### Factors associated with pain relief

As previously mentioned, few observational studies identified factors associated with greater pain relief using multivariable analysis, which restricts our ability to compare our results with those of other studies. In the present study, participants who lived with chronic post-surgical pain were less likely to experience pain relief (independently from evidence of neuropathic pain). It is important to highlight that chronic post-surgical pain represents a significant and often overlooked clinical problem (54) affecting a important proportion of patients who undergo surgery (10% to 60%) (55, 56). This condition has been associated with impaired physical function and reduced quality of life (57, 58), as well as poor mental health, general health status, sleep outcomes (59), and functional impairment (58). A literature review (*n* = 16) led to the conclusion that there is a limited body of research specifically addressing the management of chronic post-surgical pain (60). Limited by our inability to compare our results with those of other similar studies, we could hypothesize that chronic post-surgical pain is more challenging to control than other types of chronic pain or that pain relief is lower because of underlying medical conditions not resolved by the surgery. Psychological factors (e.g., surgical-induced trauma) could also be a research avenue.

Our results highlight that participants who achieved greater pain relief had an increased likelihood of reporting stressful events as circumstances surrounding the onset of pain (e.g., a trauma, a car accident, or financial difficulties perceived by the participant as stressful). It is possible that these individuals were healthy before the accident or trauma, so they may have had fewer underlying health issues than others, which could explain why they recover better. However, this result still requires further exploration in future studies.

The duration of CP experienced by patients varies and is directly proportional to the extent of the suffering and poorer outcomes of treatment (61, 62). Pagé et al. (63) reported that longer pain duration was significantly associated with a higher likelihood of reporting worsened pain. However, our results demonstrated that, independently from pain intensity, living with pain for more than ten years was associated with higher chances of achieving extensive pain relief. This result is non-intuitive as in clinical treatment settings, patients living with pain for a long time are often treatment resistant (64, 65). Unfortunately, our data do not allow for a deeper exploration of the mechanics of this association, but our hypothesis to explain this result revolves around psychosocial factors influencing the experience of chronic pain (e.g., acceptance, self-efficacy). In fact, the literature points out that longer pain duration can be associated with better self-efficacy (66), an important predictor of less functional impairment, affective distress, and severe pain (67). Self-efficacy–impairment associations are even moderated by pain duration, with larger effect sizes observed in studies involving patients living with pain for a longer time (67). The association between pain duration and pain relief found in our analysis must, however, be thoroughly investigated in future studies before drawing premature conclusions.

Pain intensity is also a crucial component of CP assessment according to IMMPACT guidelines (29, 30, 35) and potential intersecting factor. Numerous studies have established the relationship between severe pain intensity and worse CP outcomes, such as anxiety symptoms (68), high healthcare utilization and pain catastrophizing (69), and poor quality of life (70). In the present study, it was not surprising that mild pain intensity was associated with reports of greater pain relief. This result aligns with previous research that has also demonstrated a significant overall correlation between pain intensity and pain relief measurements (71–73). It is noteworthy that these findings do not imply that pain intensity should be the primary focus to achieve greater pain relief. In fact, prioritizing the improvement of other co-occurring issues, such as sleep, mood, and function, may lead to higher levels of pain relief (74). Although treatment adequacy is sometimes defined based on pain intensity scores (27, 75), we suggest measuring these two elements separately because our study demonstrates that pain intensity is associated with, but does not fully explain, the extent of pain relief. Further studies should explore the intersections of various pain qualities in relation to pain relief.

In this study, participants experiencing catastrophic thinking (i.e., feeling that the pain is terrible and will never improve) were less likely to achieved moderate to substantial or extensive pain relief. While the standard measurement of catastrophizing is the 13-item Pain Catastrophizing Scale (45), it should be noted that we used a single-item question referred to as “catastrophizing” in the NIH Minimal Dataset for Chronic Low Back Pain (46) and STarT Back Screening Tool (47). Pain catastrophizing is an important factor to consider when dealing with the psychological components of pain (69). It is among the most important predictors of poor outcomes in CP samples (76). Although our study does not allow us to establish the direction of this association (whether catastrophizing hinders pain relief or not being relieved worsens catastrophic thinking), catastrophizing is a modifiable factor that could potentially be prioritized for better pain management.

The use of prescribed pain medications was found to be significantly associated with greater pain relief. While more than 60% of persons living with CP turn to medications for pain relief (20, 21), this treatment option is considered to provide limited efficacy (14, 15). In our study, we adjusted for the use of physical or psychological treatments. This finding may indicate that, regardless of other factors, prescribed medications have an important place in the toolkit of a multimodal approach to pain management for some individuals. It is important to note that our study was conducted among a cohort of prevalent prescribed pain medication users, who probably continue to use their medication because they appreciate the balance between benefits and risks (reducing their susceptibility to adverse effects). Therefore, participants in our study who use prescribed medications likely derive benefits from their use. Our study is, however, a first exploratory step. Further studies should conduct an analysis that includes and differentiates the specific types of pharmacological, interventional (e.g., injections), physical, and psychological treatments in the analysis.

On the other hand, the use of over-the-counter pain medications was found to be associated with a decreased likelihood of reporting extensive pain relief. A possible explanation is that patients who are not relieved are seeking solutions, which is why they use over-the-counter medications. These unmet needs could be a reflection of a lack of access to care or suboptimal care. In fact, persons living with CP often resort to self-medication using over-the-counter analgesics to alleviate their symptoms (77). While over-the-counter analgesics are generally deemed safe for most adults when used according to package instructions, the literature suggests that persons with lower levels of education, more severe pain, or recurrent or persistent pain are more prone to exceed the recommended daily dosage (78). Over-the-counter pain medications, without personalized follow-up by a healthcare professional may potentially do more harm than good. Further studies could delve into the extent to which persons living with CP are well supported in their use of over-the-counter medications.

Unsurprisingly, the use of nonpharmacological pain treatments (physical or psychological) was found to be associated with an increased likelihood of reporting moderate to substantial pain relief. Many are publicly or privately accessible in the Canadian context, or can be used freely as self-management strategies (79). Such treatments are known to provide pain relief and are well tolerated (80–85).

In our study, it was observed that participants who reported having access to a trusted healthcare professional for pain management reported greater pain relief. Previous research has emphasized the significance of the relationship between healthcare professionals and their patients, recognizing it as a crucial factor in therapeutic outcomes (86, 87). This relationship encompasses various aspects, including trust, which can impact continuity of care, treatment adherence, and the willingness to seek healthcare (86). Additionally, agreement on diagnosis and treatment plans are known as variables associated with better patient satisfaction, mental health, social function and vitality (88).

In this study, greater self-perceived general health was associated with an increased likelihood of reporting extensive pain relief. Also, we found that higher levels of psychological distress were associated with a lower likelihood of achieving greater pain relief. These results are consistent with numerous studies suggesting that improving overall well-being may contribute to alleviating CP (89–92). Additionally, it is well established that psychological factors such as depression and anxiety are predictors of poorer pain-related health outcomes (93–96).

Polypharmacy (concomitant use of ≥5 medications) is a common clinical consequence of multimorbidity (97–99). For pain management, multimodal analgesia is common. It consists in the administration of multiple medications, each with a unique mechanism of action, which, when taken concurrently, can provide adequate pain relief (100). In the present study, participants with polypharmacy were more likely to achieve moderate to substantial pain relief than those without polypharmacy. Polypharmacy can lead to potential problems, such as an increased risk of drug-related adverse events, drug interactions (101, 102) and drug cascades. Still, it can be “rational” (103) and lead to positive clinical outcomes by approaching diseases through multiple mechanisms of action (104). For example, given the highly contentious nature of opioid prescribing and the frequent instances of forced withdrawal and insufficient pain relief experienced by patients (22, 105), rational polypharmacy presents an alternative approach (combining medications to address the multifaceted aspects of CP while minimizing their risks). That said, further studies should explore the specific medications used by participants in the COPE Cohort to support this hypothesis.

Although the literature is abundant with evidence demonstrating gender differences in pain “experience and treatment” (106), no association was found between gender identity or gender-stereotyped personality traits and the level of pain relief in our study (univariable or multivariable analyses).

### Strengths and limitations

While the web-based self-reported questionnaire provides no control over the real underlying condition compared to in-person assessments, the web survey approach enabled the recruitment of a large sample without geographical limitations (remote and non-remote regions). COPE Cohort participant characteristics have been shown to be similar to randomly selected samples of Canadians living with CP in terms of age, employment status, educational level, and pain characteristics (28). Furthermore, the study employed a multivariable approach to examine the relationships between numerous factors that had not been previously explored. However, at this level, our study should be considered exploratory due to its cross-sectional nature, which does not allow for establishing causal relationships. Also, despite the diversity and inclusiveness of the variables considered in the study survey, not all relevant factors were included in the questionnaire, particularly regarding information on social support, clinically meaningful relief, satisfaction with treatment or the types of medication used, dosage, and purpose (for pain or other conditions). Other aspects (e.g., economic considerations, pain meanings, beliefs, fear, avoidance) could also be relevant and even deepened using qualitative interviews. More advanced statistical approaches (e.g., interaction testing, mediation analysis, machine learning) could also be relevant to deepen our understanding of the underlying modulators of CP and pain relief. Finally, there was a higher representation of women in our sample [in random samples of Canadians with CP, women typically represent 55%–65% of the sample (28)]. This could be explained by the web-based recruitment method, as women are known to use social media more frequently and work in front of a computer more often (107). Additionally, women generally consume more medication than men (108). However, this was circumvented by the use of stratification and multivariable analysis.

## Conclusion

Based on our results, it is possible, depending on various health factors, to achieve pain relief with treatment. Individuals in this situation can provide valuable insights into their pain management strategies. The identification of factors associated with greater pain relief enabled us to explore potential opportunities for improving the well-being of individuals living with CP. For example, our results reiterate the importance of implementing a comprehensive and personalized approach to pain treatment, which integrates pharmacological, physical, and psychological interventions, along with the involvement of a trusted healthcare provider. Additionally, individuals with CP need better support if they feel the need to use over-the-counter pain medications. Improving overall well-being by addressing psychological distress and general health should also be a focal point for healthcare professionals who assist individuals living with CP. Other factors listed in the above paragraphs should also be considered in further studies, and analytical approaches should allow for exploring their intersections.

## Data Availability

The datasets presented in this article are not readily available because COPE Cohort participants did not initially provide consent to open data. The data that support the findings of this study are available from the corresponding author upon reasonable request and conditionally to a proper ethical approval for a secondary data analysis. Programming codes can be obtained directly from the corresponding author. Requests to access the datasets should be directed to lacassea@uqat.ca.
