# Supplementary material for: Pain relief and associated factors: a cross-sectional observational web-based study in a Quebec cohort of persons living with chronic pain
Source: Front Pain Res (Lausanne). 2024 Mar 15;5:1306479. doi: 10.3389/fpain.2024.1306479 (PMC10978597; doi:10.3389/fpain.2024.1306479)
Supplement: Supplementary file 1 [file Datasheet1.pdf]

Appendix 1 – COPE Cohort questionnaire

Lacasse, A., Gagnon, V., Nguena Nguefack, H. L., Gosselin, M., Pagé, M. G., Blais, L., & Guénette, L. (2021). Chronic pain patients' willingness to share personal identifiers on the web for the linkage of medico-administrative claims and patient-reported data: The chronic pain treatment cohort. *Pharmacoepidemiology and drug safety*, 30(8), 1012–1026. https://doi.org/10.1002/pds.5255

Description of variables and validated measurement scales included in the COPE Cohort web-based questionnaire.

Variables	Measurement	Sections of the questionnaire
	Chronic pain characteristics and interference †	
Location	- Semi closed-ended question *	1
Circumstances surrounding onset	- Semi closed-ended question *	1
Duration	- Open-ended question about the number of days, months or years since the onset of pain (allowing the assessment of chronic pain presence) *	1
Frequency	- Closed-ended question *	1
Intensity	- 11-point (i.e., 0–10) numerical rating scales ‡ – On average in the past 7 days/At its worst in the past 7 days *	1
Tendency to pain catastrophizing	- Closed-ended question : Agreement with the statement "*I feel that my pain is terrible and it's never going to get any better*" §	1
Neuropathic component	- Neuropathic pain questionnaire (DN4) – Interview part (Bouhassira et al., 2005) (a score >3/7 indicates a likely presence of a neuropathic component to the patient's pain; when some items were filled (yes=1 point), items not filled in were considered as 0 points (Timmerman et al., 2017)) *	2
Interference	- Brief Pain Inventory (BPI) – Interference scale (Cleeland, 2009) *‡	3
	Pain treatment and healthcare	
Section of the questionnaire designed to obtain consent for data linkage	- Free and informed consent form specific to data linkage (yes/no, reasons of refusal)<br>- Space to provide personal identifiers if linkage with public medical/drug insurance claims (health insurance number, first name, last name, date of birth)<br>- Private drug insurance information if applicable	4
Pharmacological pain treatments	- Closed-ended questions about current use of prescribed pain medications (yes/no) and over-the-counter pain medications (yes/no).<br>- Will be complemented by prescription claims.	5

Adverse effects of pharmacological pain treatments †	- Standardized checklist of adverse effects related to pain treatment evaluated in terms of presence and intensity (mild, moderate, severe) ‖	5
Non-pharmacological pain treatments	- Closed-ended question about current use of non-pharmacological pain treatments (yes/no)<br>- Semi closed-ended question about the type of treatments that are used. Listed treatments were inspired by the work of the Canadian Agency for Drugs and Technologies in Health (CATH, 2018) and the Quebec Pain Registry (Choiniere et al., 2017)	5
Percentage of relief provided by pain treatments	- Numeric scale ranging from 0% (no relief) to 100% (complete relief) adapted from the Brief Pain Inventory (BPI) (Cleeland, 2009). The BPI version only covered the past 24 hours so it was adapted to cover general relief provided by current use of pain treatments.	5
Most effective treatment	- Open-ended question	5
Access to a trusted healthcare professional for pain management	- Closed-ended question with the following examples: physician, nurse, pharmacist, physiotherapist, psychologist	5
	Sociodemographic profile	
Age *, gender identity *, race/ethnicity, country of birth, employment *, involvement in litigation related to a disability benefit claim, education level, region of residence	- Open-ended, closed-ended and semi closed-ended questions	6
Gender (gender-stereotyped personality traits) *	- Gender roles scale: Bem Sex-Role Inventory (BSRI) (Bem, 1974) – 18-item French version (Fontayne et al., 2000)	6
	Health profile	
Health-related quality of life †	- 3 items of the SF-12v2 Health Survey (SF-12v2) (Maruish, 2012) allowing the norm-based scoring of 2 of the 8 SF-12v2 subscales, i.e., Physical Functioning (PF) * and General Health (GH)	7
Polypharmacy	- Closed-ended question about the number of medications currently used (including prescribed, over-the-counter, pain-related and other diseases-related medications)	7
Emotional functioning †	- Anxiety and depressive symptoms measured by the Patient Health Questionnaire - 4 items (PHQ-4) (Kroenke et al., 2009) *	7

Smoking, alcohol and drugs	- Closed-ended questions §	7
Cannabis use	- Closed-ended questions about past year use of cannabis for pain management (yes/no), management of other health problems (yes/no), recreational purposes (yes/no)	7
Obesity	- Open-ended question about weight and height §	7

Table footnotes:
* Included in the minimum dataset suggested by the Canadian Registry Working Group of the Strategy for Patient-Oriented Research (SPOR) Chronic Pain Network (CPN) (CPN, 2017)
† Core outcome domains recommended by the Initiative on Methods, Measurement, and Pain Assessment in Clinical Trials (IMMPACT) (Turk et al., 2003)
‡ Validated scales recommended by the Initiative on Methods, Measurement, and Pain Assessment in Clinical Trials (IMMPACT) (Dworkin et al., 2005)
§ Items from the Canadian Minimum dataset for chronic low back pain research (Lacasse et al., 2017)
|| Items from the Quebec Pain Registry (Choiniere et al., 2017)

References

Bem, S. L. (1974). The measurement of psychological androgyny. *Journal of consulting clinical psychology*, *42*(2), 155-162.

Bouhassira, D., Attal, N., Alchaar, H., Boureau, F., Brochet, B., Bruxelle, J., Cunin, G., Fermanian, J., Ginies, P., Grun-Overdyking, A., Jafari-Schluep, H., Lanteri-Minet, M., Laurent, B., Mick, G., Serrie, A., Valade, D., & Vicaut, E. (2005). Comparison of pain syndromes associated with nervous or somatic lesions and development of a new neuropathic pain diagnostic questionnaire (DN4). *Pain*, *114*(1-2), 29-36. https://doi.org/10.1016/j.pain.2004.12.010 (NOT IN FILE)

CADTH. (2018). *Access to and Availability of Non-Pharmacological Treatments for Chronic Non-Cancer Pain in Canada: An Environmental Scan*. Canadian Agency for Drugs and Technologies in Health (CADTH). https://www.cadth.ca/access-and-availability-non-pharmacological-treatments-chronic-non-cancer-pain-canada-environmental

Choiniere, M., Ware, M. A., Page, M. G., Lacasse, A., Lanctot, H., Beaudet, N., Boulanger, A., Bourgault, P., Cloutier, C., Coupal, L., De Koninck, Y., Dion, D., Dolbec, P., Germain, L., Martin, V., Sarret, P., Shir, Y., Taillefer, M. C., Tousignant, B., . . . Truchon, R. (2017). Development and Implementation of a Registry of Patients Attending Multidisciplinary Pain Treatment Clinics: The Quebec Pain Registry. *Pain Res Manag*, *2017*, 8123812. https://doi.org/10.1155/2017/8123812

Cleeland, C. S. (2009). *The Brief Pain Inventory User Guide*. The University of Texas MD Anderson Cancer Center. https://www.mdanderson.org/content/dam/mdanderson/documents/Departments-and-Divisions/Symptom-Research/BPI_UserGuide.pdf

CPN. (2017). *Chronic Pain Network Annual Report 2016/2017*. https://cpn.mcmaster.ca/docs/default-source/annual-reports/2017-annual-report-en.pdf?sfvrsn=2d511708_4

Dworkin, R. H., Turk, D. C., Farrar, J. T., Haythornthwaite, J. A., Jensen, M. P., Katz, N. P., Kerns, R. D., Stucki, G., Allen, R. R., Bellamy, N., Carr, D. B., Chandler, J.,

Cowan, P., Dionne, R., Galer, B. S., Hertz, S., Jadad, A. R., Kramer, L. D., Manning, D. C., . . . Witter, J. (2005). Core outcome measures for chronic pain clinical trials: IMMPACT recommendations. *Pain*, *113*(1-2), 9-19. http://ezproxy.usherbrooke.ca/login?url=http://search.ebscohost.com/login.aspx?direct=true&db=mnh&AN=15621359&site=ehost-live

Fontayne, P., Sarrazin, P., & Famose, J.-P. (2000). The Bem Sex-Role inventory: Validation of a short version for French teenagers. *European Review of Applied Psychology/Revue Européenne de Psychologie Appliquée*, *50*(4), 405-416.

Kroenke, K., Spitzer, R. L., Williams, J. B., & Lowe, B. (2009). An ultra-brief screening scale for anxiety and depression: the PHQ-4 [Evaluation Studies

Research Support, Non-U.S. Gov't]. *Psychosomatics*, *50*(6), 613-621. https://doi.org/10.1176/appi.psy.50.6.613

Lacasse, A., Roy, J. S., Parent, A. J., Noushi, N., Odenigbo, C., Page, G., Beaudet, N., Choiniere, M., Stone, L. S., & Ware, M. A. (2017). The Canadian minimum dataset for chronic low back pain research: a cross-cultural adaptation of the National Institutes of Health Task Force Research Standards. *CMAJ Open*, *5*(1), E237-E248. https://doi.org/10.9778/cmajo.20160117

Maruish, M. E. (2012). *User's manual for the SF-12v2 Health Survey* (3rd ed.). QualityMetric Incorporated.

Timmerman, H., Steegers, M. A. H., Huygen, F., Goeman, J. J., van Dasselaar, N. T., Schenkels, M. J., Wilder-Smith, O. H. G., Wolff, A. P., & Vissers, K. C. P. (2017). Investigating the validity of the DN4 in a consecutive population of patients with chronic pain. *PLoS One*, *12*(11), e0187961. https://doi.org/10.1371/journal.pone.0187961

Turk, D. C., Dworkin, R. H., Allen, R. R., Bellamy, N., Brandenburg, N., Carr, D. B., Cleeland, C., Dionne, R., Farrar, J. T., Galer, B. S., Hewitt, D. J., Jadad, A. R., Katz, N. P., Kramer, L. D., Manning, D. C., McCormick, C. G., McDermott, M. P., McGrath, P., Quessy, S., . . . Witter, J. (2003). Core outcome domains for chronic pain clinical trials: IMMPACT recommendations. *Pain*, *106*(3), 337-345. http://ezproxy.usherbrooke.ca/login?url=http://search.ebscohost.com/login.aspx?direct=true&db=mnh&AN=14659516&site=ehost-live

LABORATOIRE DE RECHERCHE
EN ÉPIDÉMIOLOGIE DE LA
DOULEUR CHRONIQUE

Mieux comprendre la douleur chronique et son traitement

Mot d'introduction et consentement

Projet de recherche

Mieux comprendre la douleur chronique et son traitement

Chercheure principale : Anaïs Lacasse, Ph.D., chercheure au Département des sciences de la santé de l'Université du Québec en Abitibi-Témiscamingue (UQAT)

Équipe de recherche : Véronique Gagnon, Inf., étudiante à la maîtrise recherche en sciences de la santé à l'UQAT ; Gabrielle Pagé, Ph.D., chercheure au Centre de recherche du Centre hospitalier de l'Université de Montréal ; Lucie Blais, Ph.D., chercheure à la Faculté de pharmacie de l'Université de Montréal ; Lyne Guénette, Ph.D., chercheure à la Faculté de pharmacie de l'Université Laval

Le présent projet de recherche vise à mieux comprendre la douleur chronique et son traitement. Vous êtes donc invité(e) à participer à cette étude menée par la professeure Anaïs Lacasse et son équipe. Ce projet de recherche a été financé par le Réseau québécois de recherche sur les médicaments (RQRM). Il a aussi été approuvé par le Comité d'éthique de la recherche de l'Université du Québec en Abitibi-Témiscamingue.

Pour être admissible, vous devez : 1) souffrir de douleur de façon constante ou occasionnelle depuis plus de 3 mois (peu importe si cette douleur est légère ou sévère et peu importe la cause), 2) être âgé(e) de 18 ans ou plus, 3) habiter au Québec, 4) être capable de répondre à un questionnaire en français.

Votre participation à ce projet de recherche consiste à consacrer environ 20 minutes de votre temps pour remplir notre questionnaire en ligne. Pendant que vous remplissez le questionnaire, vous pouvez prendre une pause, mais prière de laisser votre navigateur web ouvert afin de ne pas perdre les réponses déjà entrées.

Vous n'avez pas d'avantage immédiat relié à votre participation à cette étude et aucune indemnité compensatoire ne vous sera accordée. Toutefois, nos résultats contribueront à mieux comprendre l'usage, les bénéfices et les risques des traitements pour soulager la douleur chronique en contexte réel de pratique clinique. Le seul inconvénient découlant de votre participation, s'il en est, est le temps que vous consacrerez à remplir notre questionnaire en ligne.

Tous les renseignements obtenus sur vous dans le cadre de ce projet de recherche demeureront confidentiels. Pour éviter tout risque lié à la sécurité de vos données, prière d'éviter de répondre à ce questionnaire sur un réseau sans fil (WI-FI) public (ex. dans un café, aéroport, bibliothèque, etc.). Si vous travaillez sur un ordinateur partagé, il est aussi suggéré d'effacer votre historique de navigation.

Notre équipe de recherche affirme ne pas être en conflit d'intérêts réel, éventuel ou apparent avec le présent projet et ne pas viser aucune commercialisation des résultats.

Nous tenons à vous assurer que votre participation à cette étude est volontaire et que vous pouvez, en tout temps, sauter certaines questions ou arrêter de remplir notre questionnaire en ligne. En acceptant de participer à cette étude, vous ne renoncez à aucun de vos droits ni ne libérez les chercheurs ou le commanditaire de leurs obligations légales et professionnelles à votre égard. Si vous en faites la demande à la fin du questionnaire, il vous sera possible de recevoir un résumé des résultats par courriel.

Toute question concernant le projet pourra être adressée à Professeure Anaïs Lacasse : Douleur@uqat.ca / 1 877 870-8728, poste 2722

Pour tout renseignement supplémentaire concernant vos droits, vous pouvez vous adresser au :
Comité d’éthique de la recherche avec des êtres humains de l’Université du Québec en Abitibi-Témiscamingue
Vice-rectorat à l’enseignement et à la recherche
445, boulevard de l’Université, bureau B-309
Rouyn-Noranda (Québec) J9X 5E4
1 877 870-8728, poste 2252
cer@uqat.ca

*** J’accepte de participer au sondage et je confirme être âgé(e) de 18 ans et plus et habiter au Québec**

- ◯ Oui
- ◯ Non

Veuillez conserver un exemplaire de cette page pour vos dossiers

LABORATOIRE DE RECHERCHE EN ÉPIDÉMIOLOGIE DE LA DOULEUR CHRONIQUE

Mieux comprendre la douleur chronique et son traitement

Merci quand même pour votre intérêt!

Merci quand même pour votre intérêt!
Afin de savoir à quel point nos participants sont représentatifs de l'ensemble des personnes ayant entendu parler de notre sondage, nous vous invitons à répondre aux 3 questions suivantes :

Âge (18-120) :

Sexe :

◯ Femme ◯ Homme ◯ Inconnu ◯ Indéterminé

Raison(s) du refus?

UQAT
LABORATOIRE DE RECHERCHE EN ÉPIDÉMIOLOGIE DE LA DOULEUR CHRONIQUE

Mieux comprendre la douleur chronique et son traitement

Caractéristiques de la douleur

*** Dans quelle(s) région(s) du corps ressentez-vous de la douleur?**

(cochez toutes les cases applicables)

- ☐ Douleurs généralisées dans tout le corps (ou presque)
- ☐ Tête
- ☐ Visage
- ☐ Cou
- ☐ Épaule(s)
- ☐ Bras
- ☐ Coude(s)
- ☐ Poignet(s)
- ☐ Main(s)
- ☐ Haut du dos
- ☐ Bas du dos
- ☐ Autre (veuillez préciser)
- ☐ Poitrine
- ☐ Abdomen / Estomac
- ☐ Hanche(s)
- ☐ Fesse(s)
- ☐ Région anale
- ☐ Région génitale
- ☐ Jambe(s)
- ☐ Genou(x)
- ☐ Cheville(s)
- ☐ Pied(s)

Si vous souffrez de douleur dans plus d'une région du corps, veuillez répondre à toutes les questions suivantes en tenant compte de l'endroit <u>qui fait le plus mal</u>

*** Indiquez les circonstances qui, selon vous, ont mené à l'apparition de votre douleur (cochez plus d'une réponse s'il y a lieu)**

- ☐ Accident au travail
- ☐ Accident à la maison
- ☐ Accident avec véhicule motorisé
- ☐ Accident de sport
- ☐ À la suite d’une chirurgie
- ☐ À la suite d’un événement stressant
- ☐ Postures ou mouvements répétitifs
- ☐ Durant ou à la suite d’un cancer
- ☐ Durant ou à la suite d’une maladie (autre que le cancer)
- ☐ Maladie dégénérative liée à l’âge (ex. arthrose)
- ☐ Maladie inflammatoire ou auto-immune (ex. arthrite rhumatoïde, lupus)
- ☐ Aucun événement précis
- ☐ Je ne sais pas
- ☐ Autre raison ou événement (veuillez préciser)

Est-ce que votre douleur est :

- ○ Présente continuellement
- ○ Présente occasionnellement

Depuis combien de temps ressentez-vous votre douleur?

En jours :

OU

En mois :

OU

En années :

Intensité de la douleur

	0 (AUCUNE DOULEUR)	1	2	3	4	5	6	7	8	9	10 (LA PIRE DOULEUR POSSIBLE)
Veuillez choisir sur l'échelle suivante le chiffre qui décrit le mieux la pire douleur que vous avez ressentie au cours des 7 derniers jours	◯	◯	◯	◯	◯	◯	◯	◯	◯	◯	◯
Veuillez choisir sur l'échelle suivante le chiffre qui décrit le mieux la douleur que vous avez ressentie en moyenne ou en général au cours des 7 derniers jours	◯	◯	◯	◯	◯	◯	◯	◯	◯	◯	◯

Êtes-vous en accord avec l'énoncé suivant :
Je considère que ma douleur est épouvantable et j'ai l'impression que cela ne s'améliorera jamais.

◯ D'accord

◯ Pas d'accord

LABORATOIRE DE RECHERCHE EN ÉPIDÉMIOLOGIE DE LA DOULEUR CHRONIQUE

Mieux comprendre la douleur chronique et son traitement

Composante neuropathique de la douleur (questionnaire DN4)

Si vous souffrez de douleur dans plus d'une région du corps, veuillez répondre à toutes les questions suivantes en tenant compte de l'endroit qui fait le plus mal

La douleur présente-t-elle une ou plusieurs des caractéristiques suivantes ?

	Oui	Non
Brûlure	○	○
Sensation de froid douloureux	○	○
Décharges électriques	○	○

La douleur est-elle associée dans la même région à un ou plusieurs des symptômes suivants?

	Oui	Non
Fourmillements	○	○
Picotements	○	○
Engourdissement	○	○
Démangeaisons	○	○

UQAT
LABORATOIRE DE RECHERCHE EN ÉPIDÉMIOLOGIE DE LA DOULEUR CHRONIQUE

Mieux comprendre la douleur chronique et son traitement

Interférence de la douleur

Veuillez choisir sur les échelles suivantes le chiffre qui décrit le mieux comment, <u>au cours des 7 derniers jours</u>, la douleur a gêné votre :

	0 (NE GÊNE PAS)	1	2	3	4	5	6	7	8	9	10 (GÊNE COMPLÈTEMENT)
Activité générale	○	○	○	○	○	○	○	○	○	○	○
Humeur	○	○	○	○	○	○	○	○	○	○	○
Capacité à marcher	○	○	○	○	○	○	○	○	○	○	○
Travail habituel (y compris le travail à l’extérieur de la maison et les travaux domestiques)	○	○	○	○	○	○	○	○	○	○	○
Relation avec les autres	○	○	○	○	○	○	○	○	○	○	○
Sommeil	○	○	○	○	○	○	○	○	○	○	○
Goût de vivre	○	○	○	○	○	○	○	○	○	○	○

N.B. si vous n’avez ressenti aucune douleur au cours des 7 derniers jours, veuillez choisir " 0 " sur les échelles ci-dessus

LABORATOIRE DE RECHERCHE EN ÉPIDÉMIOLOGIE DE LA DOULEUR CHRONIQUE

Mieux comprendre la douleur chronique et son traitement

Jumelage confidentiel de données personnelles

Nous aimerions en connaître davantage sur votre utilisation de médicaments prescrits et de soins de santé (ex. consultations dans les hôpitaux, cliniques et bureaux de médecins). Pour ce faire, nous vous demandons la permission de jumeler les données recueillies dans ce questionnaire aux données de la **Régie de l'assurance maladie du Québec (RAMQ)**. Ceci nous permettra de recevoir les informations au sujet des médicaments et des services de santé que vous avez utilisés dans les 5 dernières années et que vous utiliserez dans la prochaine année.

Ces renseignements jumelés demeureront strictement confidentiels et ne seront utilisés qu'à des fins de recherche. **Notez que vous pouvez refuser, mais répondre au reste du questionnaire.**Un refus ne modifiera en rien la qualité ou la quantité des soins ou services de santé que vous recevez ou auxquels vous avez droit.

Si vous acceptez, une personne désignée au sein de l'équipe de recherche transmettra votre prénom, nom de famille, date de naissance, sexe, numéro de carte d'assurance maladie et informations sur votre assurance à la RAMQ (après avoir obtenu l'approbation de la Comission d'accès à l'information du Québec).

Tous les transferts d'information seront effectués par courrier recommandé et fichiers informatiques sécurisés. L'équipe de recherche procédera ensuite au jumelage des bases de données. Afin de préserver votre identité et la confidentialité de vos renseignements personnels, toutes les informations permettant de vous identifier seront par la suite effacées de la banque de données et vous serez identifié(e) que par un numéro de code. Il nous serait donc impossible de détruire les données d'un participant qui en ferait la demande.

Est-ce que vous nous donnez la permission d'effectuer ce jumelage?

○ Oui ○ Non

<u>Sinon</u>, prière de nous indiquer pour quelle(s) raison(s) et passer à la page suivante

<u>Si oui</u>, prière de compléter les sections suivantes :

Prénom :

[ ]

Nom de famille :

[ ]

Numéro d'assurance maladie tel qu'il apparaît sur votre carte d'assurance maladie du Québec **(sans espaces)** :

[ ]

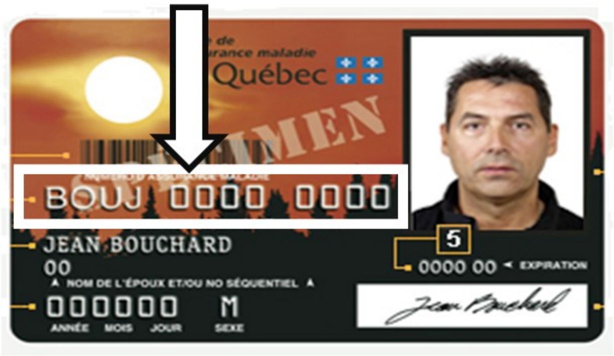

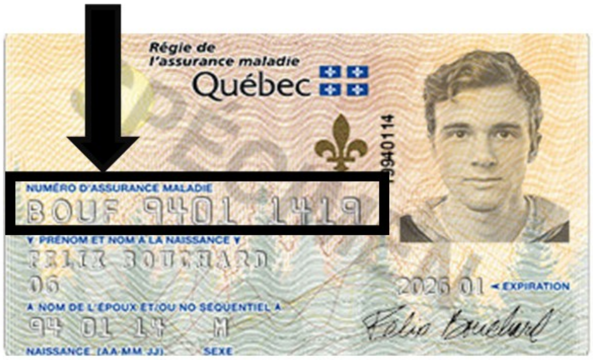

Date de naissance

Date de naissance

JJ/MM/AAAA

Pour vos médicaments sur ordonnance (médicaments prescrits), avez-vous eu à un moment ou à un autre une assurance médicaments collective ou privée au cours de la dernière année?

- ◯ Oui
- ◯ Non
- ◯ Je ne sais pas

L’assurance médicament du gouvernement (RAMQ) couvre seulement une portion de la population québécoise et il nous est impossible d’avoir accès via la RAMQ aux données liées aux médicaments achetés par les personnes qui ont une assurance médicaments collective ou privée. Un registre permettant cet accès appelé reMed est cependant maintenu par professeure Lucie Blais, collaboratrice au présent projet et chercheure à la Faculté de Pharmacie de l’Université de Montréal. Cet accès nécessite cependant un consentement séparé et une inscription au registre reMed. Accepteriez-vous que notre équipe communique avec vous par courriel ou par la poste pour mettre en place cet accès?

○ Oui

○ Non

Sinon, passez à la page suivante.

Si oui, prière d'inscrire votre adresse courriel dans la case ci-dessous :

Si vous n’avez pas d’adresse courriel, vous pouvez toujours nous laisser votre adresse postale :

Nom

Adresse

Adresse 2

Ville/Localité

Province

Code postal

Pays

LABORATOIRE DE RECHERCHE
EN ÉPIDÉMIOLOGIE DE LA
DOULEUR CHRONIQUE

Mieux comprendre la douleur chronique et son traitement

Traitement de la douleur

Pour le traitement de votre douleur, utilisez-vous actuellement des médicaments prescrits (qui nécessitent une ordonnance de la part d'un médecin, d'un pharmacien ou d'une infirmière praticienne)?

- ◯ Oui
- ◯ Non

Pour le traitement de votre douleur, utilisez-vous actuellement des médicaments en vente libre (qui ne nécessitent pas d'ordonnance, par exemple: Robax®, Tylenol®, ou Advil®)?

- ◯ Oui
- ◯ Non

Ressentez-vous l'un ou l'autre des effets secondaires suivants à cause de votre traitement actuel contre la douleur?

	Aucun	Léger	Modéré	Sévère
Vertige / étourdissement	○	○	○	○
Somnolence	○	○	○	○
Confusion	○	○	○	○
Nausée	○	○	○	○
Vomissement	○	○	○	○
Perte de mémoire	○	○	○	○
Bouche sèche	○	○	○	○
Démangeaison	○	○	○	○
Inconfort abdominal	○	○	○	○
Constipation	○	○	○	○
Ralentissement du débit urinaire	○	○	○	○
Fatigue	○	○	○	○
Insomnie	○	○	○	○
Enflure	○	○	○	○
Gain de poids	○	○	○	○
Vision brouillée	○	○	○	○
Baisse de libido	○	○	○	○
Hallucinations	○	○	○	○
Cauchemars	○	○	○	○

Autre (veuillez préciser)

Mis à part des médicaments, utilisez-vous actuellement d'autres types de traitements pour votre douleur?

○ Oui

○ Non

Si oui, lesquels (cochez toutes les réponses qui s'appliquent)?

- ☐ Acupuncture /Acupression
- ☐ Aquathérapie/Hydrothérapie (ex. bain, aqua forme)
- ☐ Aromathérapie (ex. extraits de plantes, huiles essentielles)
- ☐ Biofeedback
- ☐ Chaud et/ou froid
- ☐ Chiropratique
- ☐ Ergothérapie
- ☐ Exercice/activité physique
- ☐ Groupes de soutien/d'entraide (qui regroupe seulement des patients)
- ☐ Hypnose
- ☐ Implant d'un neurostimulateur
- ☐ Autre (veuillez préciser)
- ☐ Injections (ex. bloc ou autres techniques)
- ☐ Intervention de groupe (ex. école du dos)
- ☐ Massothérapie / Massage
- ☐ Méditation
- ☐ Musicothérapie (thérapie par la musique)
- ☐ Ostéopathie
- ☐ Physiothérapie
- ☐ Produits homéopathiques
- ☐ Produits naturels
- ☐ Psychothérapie
- ☐ Réalité virtuelle ou augmentée
- ☐ Réflexologie
- ☐ Reiki
- ☐ Stimulation nerveuse électrique transcutanée (TENS)
- ☐ Taï Chi
- ☐ Taping (bandes élastiques)
- ☐ Techniques de relaxation/respiration
- ☐ Thérapie cognitivo comportementale
- ☐ Thérapie comportementale
- ☐ Yoga
- ☐ Zoothérapie (thérapie grâce aux animaux)

Dans l'ensemble, quel soulagement les traitements ou médicaments utilisés actuellement pour votre douleur vous apportent-ils?

	0 % (AUCUN SOULAGEMENT)	10 %	20 %	30 %	40 %	50 %	60 %	70 %	80 %	90 %	100 % (SOULAGEMENT COMPLET)
Choix de réponses :	○	○	○	○	○	○	○	○	○	○	○

De tous vos traitements utilisés actuellement (incluant les médicaments, les approches non pharmacologiques, les interventions, etc.), qu'est-ce qui vous apparaît le plus efficace?

Avez-vous accès à un professionnel de la santé en qui vous avez confiance pour le traitement de votre douleur (ex. médecin, infirmière, pharmacien, physiothérapeute, psychologue, etc.)?

- ○ Oui
- ○ Non

LABORATOIRE DE RECHERCHE EN ÉPIDÉMIOLOGIE DE LA DOULEUR CHRONIQUE

Mieux comprendre la douleur chronique et son traitement

Profil sociodémographique

Âge (18-120) :

- ◯ Femme
- ◯ Homme
- ◯ Inconnu
- ◯ Indéterminé

Êtes-vous autochtone, c'est-à-dire, Première Nation, Métis ou Inuk (Inuit)?

- ☐ Non, je ne suis pas autochtone
- ☐ Oui, Première Nation - Indien(ne) de l'Amérique du Nord
- ☐ Oui, Métis
- ☐ Oui, Inuk (Inuit)

Quel est votre pays de naissance?

- ◯ Canada
- ◯ Autre (veuillez préciser)

Êtes-vous?

- ☐ Blanc/Blanche
- ☐ Sud-Asiatique - ex. Indien(ne) de l’Inde, Pakistanais(e), Sri-Lankais(e)
- ☐ Chinois(e)
- ☐ Noir(e)
- ☐ Philippin(e)
- ☐ Latino-Américain(e)
- ☐ Arabe
- ☐ Asiatique du Sud-Est - ex. Vietnamien(ne), Cambodgien(ne), Malaisien(ne), Laotien(ne)
- ☐ Asiatique occidental(e) - ex. Iranien(ne), Afghan(e)
- ☐ Coréen(ne)
- ☐ Japonais(e)
- ☐ Autre :

(veuillez préciser)

Quel statut d’emploi représente le mieux votre situation?

- ◯ Travaille présentement à temps plein
- ◯ Travaille présentement à temps partiel
- ◯ À la recherche d’emploi / sans emploi
- ◯ Congé de maladie
- ◯ Congé de maternité
- ◯ Invalidité temporaire ou permanente en raison de votre douleur
- ◯ Invalidité pour d’autres raisons que la douleur
- ◯ Étudiant
- ◯ Mise à pied temporaire (chômage planifié)
- ◯ Retraité(e)
- ◯ Au foyer
- ◯ Inconnu
- ◯ Autre :
- ◯ (veuillez préciser)

Êtes-vous actuellement impliqué(e) dans un litige pour obtenir des prestations d’invalidité ?

Par exemple avec l’employeur, un assureur, la Commission des normes, de l'équité, de la santé et de la sécurité du travail (CNESST), la Société de l’Assurance automobile du Québec (SAAQ) ou l’Indemnisation des victimes d’actes criminels (IVAC)

- ◯ Oui
- ◯ Non
- ◯ Incertain(e)

Niveau de scolarité (sélectionnez le plus haut niveau atteint) :

- ◯ Sans diplôme d’études secondaires
- ◯ Diplôme d'études secondaires ou l'équivalent
- ◯ Certificat ou diplôme d'apprenti inscrit, d'une école de métiers ou d'un centre de formation professionnelle? (ex. coiffure, cuisine, électricien, charpenterie)
- ◯ Certificat ou diplôme d'études collégiales, d'un CÉGEP ou d'un autre établissement non universitaire (ex. technique de comptabilité, technique de génie industriel, adjoint juridique, DEC préuniversitaire, etc.)
- ◯ Programme universitaire de 1er cycle (ex. baccalauréat, certificat)
- ◯ Programme universitaire de 2e cycle (ex. maîtrise, D.E.S.S., microprogramme de 2e cycle)
- ◯ Doctorat (ex. MD, PharmD, Ph.D., D.Psy., D.Ed.)

Dans quelle région du Québec habitez-vous présentement?

- Abitibi-Témiscamingue
- Bas-Saint-Laurent
- Capitale Nationale
- Centre-du-Québec
- Chaudière-Appalaches
- Côte-Nord
- Estrie
- Gaspésie-Îles-de-la-Madeleine
- Lanaudière
- Laurentides
- Laval
- Mauricie
- Montérégie
- Montréal
- Nord-du-Québec
- Outaouais
- Saguenay-Lac-Saint-Jean
- Autre (veuillez préciser)

Svp, indiquez à quel point chacune des caractéristiques ci-dessous vous représente :

	1 (jamais ou presque jamais vrai)	2	3	4	5	6	7 (presque toujours vrai)
J'ai confiance en moi							
J'aime rendre service							
Je suis sportif(ve)							

	1 (jamais ou presque jamais vrai)	2	3	4	5	6	7 (presque toujours vrai)
Je suis affectueux(se)	○	○	○	○	○	○	○
Je suis sûr(e) de moi	○	○	○	○	○	○	○
Je suis énergique	○	○	○	○	○	○	○
Je suis toujours prêt(e) à écouter les autres	○	○	○	○	○	○	○
J'ai des qualités de commandement	○	○	○	○	○	○	○
Je suis attentif(ve) aux besoins des autres	○	○	○	○	○	○	○
Je suis sensible aux peines et aux problèmes des autres	○	○	○	○	○	○	○
Je suis prêt(e) à consoler les gens	○	○	○	○	○	○	○
Je suis dominateur(trice)	○	○	○	○	○	○	○
Je suis chaleureux(se)	○	○	○	○	○	○	○
Je suis tendre	○	○	○	○	○	○	○
Je me comporte en chef	○	○	○	○	○	○	○
J'ai l'esprit de compétition	○	○	○	○	○	○	○
J'aime les enfants	○	○	○	○	○	○	○
Je suis doux(ce)	○	○	○	○	○	○	○

UQAT
LABORATOIRE DE RECHERCHE
EN ÉPIDÉMIOLOGIE DE LA
DOULEUR CHRONIQUE

Mieux comprendre la douleur chronique et son traitement

Informations générales sur votre santé

En général, diriez-vous que votre santé est :

- ◯ Excellente
- ◯ Très bonne
- ◯ Bonne
- ◯ Passable
- ◯ Mauvaise

Combien de médicaments différents utilisez-vous actuellement (qu'ils soient prescrits ou en vente libre ; qu'ils soient pour la douleur ou tout autre problème de santé)?

[ ]

Au cours des 2 dernières semaines, à quelle fréquence avez-vous été dérangé(e) par les problèmes suivants?

	Jamais	Plusieurs jours	Plus de 7 jours	Presque tous les jours
Sentiment de nervosité, d’anxiété ou de tension	○	○	○	○
Incapable d’arrêter de vous inquiéter ou de contrôler vos inquiétudes	○	○	○	○
Peu d’intérêt ou de plaisir à faire des choses	○	○	○	○
Se sentir triste, déprimé ou désespéré	○	○	○	○

Les questions suivantes portent sur les activités que vous pourriez avoir à faire au cours d'une journée normale. Votre état de santé actuel vous limite-t-il dans ces activités? Si oui, dans quelle mesure?

	Mon état de santé me limite beaucoup	Mon état de santé me limite un peu	Mon état de santé ne me limite pas du tout
Dans les <u>activités modérées</u> comme déplacer une table, passer l'aspirateur, jouer aux quilles ou au golf	○	○	○
Pour monter <u>plusieurs</u> étages à pied	○	○	○

Au cours de la dernière année...

	Jamais	Rarement	Parfois	Souvent
Avez-vous consommé de l'alcool ou des drogues plus que vous ne l'auriez voulu?	◯	◯	◯	◯
Avez-vous déjà voulu ou ressenti le besoin de réduire votre consommation d'alcool ou de drogues?	◯	◯	◯	◯

Au cours de la dernière année, avez-vous utilisé du cannabis?

	Oui	Non
Pour la gestion de votre douleur	◯	◯
Pour la gestion d'autres problèmes de santé	◯	◯
À des fins récréatives (pour le plaisir)	◯	◯

Quel énoncé décrit le mieux vos habitudes par rapport à la cigarette?

- ◯ Je n'ai jamais fumé
- ◯ Je suis un fumeur
- ◯ J'ai déjà fumé, mais je ne fume plus

Taille :

En pieds/pouces (ex. 5'6) : __________

OU

En centimètres (cm) : __________

Poids :

En livres (lbs) : [ ]

OU

En kilogrammes (kg) : [ ]

Comment avez-vous entendu parler de ce sondage?

- ◯ Facebook
- ◯ Twitter
- ◯ Instagram
- ◯ Courriel reçu de la part d'un ami, d'un membre de votre famille, d'un collègue de travail ou d'un professeur
- ◯ Courriel reçu de la part d'une association de patients (ex. Association québécoise de la douleur chronique, Association de la fibromyalgie, Société de l'arthrite)
- ◯ Site web de l'Université du Québec en Abitibi-Témiscamingue (UQAT)
- ◯ Site web d'une station de radio
- ◯ Entendu à la radio
- ◯ Journal en format papier
- ◯ Autre (veuillez préciser)

[ ]

Le questionnaire sur la douleur et son traitement se termine ici

Aimeriez-vous recevoir un résumé des résultats à la fin du projet?

◯ Oui ◯ Non

Aimeriez-vous être recontacté(e) pour de futures études réalisées par notre groupe de recherche?

◯ Oui ◯ Non

Si vous avez répondu oui à l'une ou l'autre des 2 questions précédentes, svp nous laisser votre adresse courriel dans la case ci-dessous :

[ ]

Si vous n’avez pas d’adresse courriel, vous pouvez toujours nous laisser votre adresse postale :

Nom

Adresse

Adresse 2

Ville/Localité

Province

Code postal

Pays

Merci beaucoup de votre participation!

Nous sommes très reconnaissants que vous ayez accepté de nous aider dans nos recherches!

UQAT

LABORATOIRE DE RECHERCHE
EN ÉPIDÉMIOLOGIE DE LA
DOULEUR CHRONIQUE
